# A Stem-Cell Based Bioassay to Critically Assess the Pathology of Dysfunctional Neuromuscular Junctions

**DOI:** 10.1371/journal.pone.0091643

**Published:** 2014-03-13

**Authors:** Peter H. Chipman, Ying Zhang, Victor F. Rafuse

**Affiliations:** 1 Department of Medical Neuroscience, Dalhousie University, Halifax, Nova Scotia, Canada; 2 Department of Medicine, Dalhousie University, Halifax, Nova Scotia, Canada; 3 Brain Repair Centre, Life Science Research Centre, Halifax, Nova Scotia, Canada; University of Sheffield, United Kingdom

## Abstract

Pluripotent stem cells can be directed to differentiate into motor neurons and assessed for functionality *in vitro*. An emerging application of this technique is to model genetically inherited diseases in differentiated motor neurons and to screen for new therapeutic targets. The neuromuscular junction (NMJ) is essential to the functionality of motor neurons and its dysfunction is a primary hallmark of motor neuron disease. However, mature NMJs that possess the functional and morphological characteristics of those formed *in vivo* have so far not been obtained *in vitro*. Here we describe the generation and analysis of mature NMJs formed between embryonic stem cell-derived motor neurons (ESCMNs) and primary myotubes. We compared the formation and maturation of NMJs generated by wild-type (*NCAM^+/+^*) ESCMNs to those generated by neural cell adhesion molecule null (*NCAM^-/-^*) ESCMNs in order to definitively test the sensitivity of this assay to identify synaptic pathology. We find that co-cultures using *NCAM^-/-^* ESCMNs replicate key *in vivo NCAM^-/-^* phenotypes and reveal that NCAM influences neuromuscular synaptogenesis by controlling the mode of synaptic vesicle endocytosis. Further, we could improve synapse formation and function in *NCAM^-/-^* co-cultures by chronic treatment with nifedipine, which blocks an immature synaptic vesicle recycling pathway. Together, our results demonstrate that this ESCMN/myofiber co-culture system is a highly sensitive bioassay for examining molecules postulated to regulate synaptic function and for screening therapeutics that will improve the function of compromised NMJs.

## Introduction

Several classes of motor neuron diseases (MNDs) manifest themselves as disorders of the neuromuscular junction (NMJ) prior to overt cell death [1]–[8]. Even when motor neurons are prevented from dying in a mouse model of amyotrophic lateral sclerosis (ALS), motor axons still degenerate from the motor endplate causing muscle paralysis and death [4]. This degeneration is due, at least in part, to anatomical and/or functional deficits at the NMJ. Understanding these deficits could lead to the development of therapeutics that will improve NMJ function and thus attenuate the progression of the disease.

Large scale screening of therapeutics aimed at improving the function of NMJs in MNDs requires the creation of an *in vitro* model system that accurately emulates normal development, function and long-term stability of the motor endplate *in vivo*. The model system should also exhibit the same anatomical and functional deficits as their endogenous counterparts when cellular components of the NMJ are mutated or missing. Furthermore, the *in vitro* system should be able to incorporate genetic mutations known to cause familial forms of MNDs. Finally, the structure and function of the co-cultured system should respond appropriately to pharmacological interventions.

A co-culture system consisting of motor neurons derived from embryonic stem (ES) cells and muscle fibers may achieve many of the above conditions. ES cell-derived motor neurons (ESCMNs) exhibit functional and genetic properties consistent with their *in vivo* counterparts [9]–[11] and they form immature synaptic contacts when co-cultured with myofibers [10], [12], [13]. However, it is not known to what extent this co-culture system accurately reflects normal neuromuscular development/function or whether it recapitulates the same structural and functional abnormalities seen *in vivo* when proteins involved in synaptic function are absent.

To examine these issues we co-cultured ESCMNs lacking neural cell adhesion molecule (NCAM) with primary muscle fibers. We focused on NCAM because mice lacking NCAM have well described structural and functional deficits at the NMJ [14]–[17]. Furthermore, because MNDs are not embryonic lethal, we chose a mutation that alters the structure and function of the NMJ, but does not prevent its development. Thus, while *NCAM^-/-^* mice are viable [18], they have smaller NMJs [16], [17], abnormal synaptic vesicle dynamics [19]–[21], lower quantal content at reinnervated endplates [22], and highly disordered intramuscular motor axon branching [23]. Furthermore, although NCAM is expressed by motor neurons, muscle fibers, and terminal Schwann cells [24], [25], most abnormalities at the NMJ are likely due to the loss of presynaptic NCAM [20], [21], [26].

Here we report that *NCAM^-/-^* ESCMN/myofiber co-cultures exhibit the same phenotypes observed in *NCAM^-/-^* mice. Moreover, we demonstrate that compromised neurotransmission and synaptic growth exhibited by *NCAM^-/-^* ESCMNs can be relieved by inhibiting L-type voltage dependent calcium channel (L-VDCC) mediated synaptic vesicle recycling. Thus, ESMN/muscle fiber co-culture system is a very sensitive bioassay for examining molecules postulated to regulate synaptic function and for screening therapeutics that will improve function at compromised NMJs.

## Methods

### Generation and differentiation of embryonic stem cells

Stem cells from two transgenic mouse lines were used in this study. HBG3 mouse ES cells [9] (a kind gift provided by Dr. T. Jessell, Columbia University, New York NY) were originally derived from Hb9:GFP transgenic mice [27] (Jackson Labs, Bar Harbour, Maine) and used as wild-type controls (referred throughout as *NCAM^+/+^*). Hb9:GFP mice were bred with *NCAM^-/-^* mice (generated on a C57/Bl6 background) to generate Hb9:GFP *NCAM^-/-^* ES cells (referred throughout as *NCAM^-/-^*), which were isolated from the inner cell mass of a mouse blastocysts using standard techniques. Briefly, pregnant females were sacrificed on the 3^rd^ day of pregnancy when embryos are at the 8–16 cell stage. The uterine horn was extracted and placed in warmed M2 media (Sigma M7167). Blastocyts were flushed from the uterine horn and transferred to a 4 well plate containing a confluent monolayer of mitomycin C treated (Sigma) primary mouse embryonic fibroblasts (PMEFs). The inner cell mass (ICM) was allowed to expand for approximately 4 days then fed every two days with embryonic stem cell media (ESC media) containing DMEM (Gibco 11995-073), ESC grade fetal bovine serum (FBS; 15% by volume; Millipore ES-009-B), penicillin/streptomysin (1% by volume; Gibco 5140-122), 2-mercaptoethanol (1% by volume; Millipore ES-007-E), non-essential amino acids (1% by volume; Millipore TMS-001-C) and ESGRO LIF (leukaemia inhibitory factor; 1000 u/ml; Millipore ESG1106). After another 4–6 days, the ICM was mechanically separated from the blastocyst using a tungsten needle and 2.5% trypsin. Cells were further dissociated into small clumps and transferred onto a 4 well plate containing a confluent monolayer of PMEFs and ESC media where they were allowed to expand into ES colonies and were passaged as needed to avoid confluence. This cell line was generated at Dalhousie University with the approval of the Dalhousie University Committee on Laboratory Animals.

Isolated ES cell colonies were differentiated into motor neurons as described previously [10], [11]. In brief, ES cells were grown as aggregate cultures in DFK-10 media to form free floating embryoid bodies. DFK10 medium consisted of DMEM (Gibco 11995-073) and Ham's F-12 media (Specialty Media) in a 1∶1 ratio supplemented with knock-out serum replacement (10% by volume; Invitrogen, Burlington, Ontario, Canada), penicillin/streptomycin (1% by volume; Sigma, St. Louis, MO), N2 supplement (2.4% by volume; Invitrogen), glucose (4500 mg/l), L-glutamine (200 mM), heparin (1 u/l; Sigma), and β-mercaptoethanol (0.1 mM; Sigma). After 2 days, the embryoid bodies were treated with a smoothened agonist and RA (1M; Sigma, St. Louis, MO) and cultured as free-floating cells for an additional 5 days. GFP expression was monitored as an assessment of differentiation and only embryoid bodies with robust and homogenous GFP expression were used for further assessment.

All procedures in this study were conducted in accordance with the guidelines of the Canadian Council on Animal Care and specifically approved by the Dalhousie University Committee on Laboratory Animals.

### Co-culture of embryonic stem cell derived motor neurons and embryonic chick myotubes

Embryoid bodies containing embyronic stem cell derived motor neurons (ESCMNs) were plated on chick myotube cultures for analysis of NMJs. Muscles were dissected from stage 38 White leghorn chicks and mechanically dissociated in Ham's F-10 containing 10% horse serum (Invitrogen), 5% chicken embryo extract and 1.26 mM CaCl_2_. 10^5^ myoblasts were plated on coverslips in 24-well cell culture plates and fed after 2 days in culture with Ham's F-10 media as described above. Chick myoblasts were grown for three days before plating ESCMNs to allow for myotube fusion. In some cases, cells were treated after 2 days with cytosine β-D-arabinofuranoside (5 μM; Sigma) to remove the fibroblasts from the culture. One hour prior to ESMN plating, F-10 media was replaced with Neurobasal (Invitrogen) supplemented with 2 mM L-glutamine (Invitrogen), penicillin/streptomycin (Invitrogen), B27 Supplement (Invitrogen), GDNF (glial-derived neurotrophic factor) (20 ng/ml; Upstate Biotechnology, Lake Placid, NY) and CNTF (ciliary neurotrophic factor) (10 ng/ml; Upstate Biotechnology). Co-cultures were fed every 2 days for up to a week with neurotrophic supplemented Neurobasal formulation. For co-cultures grown for longer periods, neurotrophic factors were omitted from the Neurobasal formulation.

### Immunofluorescence and imaging

Cells were fixed with 3.7% formaldehyde (Fisher Scientific, Houston, TX) for 15 minutes at room temperature, washed with PBS and incubated in 0.1 M glycine/PBS for 1 hour. Cells were then incubated overnight at room temperature in cocktails of primary antibodies as described in Table 1. Cultures were washed thoroughly in PBS and incubated for 1 hour in a corresponding cocktail of secondary antibodies as described in Table 2. Antibodies were applied in the presence of 10% blocking solution and 0.3% TritonX-100 in PBS. For experiments requiring only surface antigen labeling, 0.3% TritonX-100 was omitted from the labeling solution. Some cells were incubated in a PBS solution containing tetramethylrhodamine conjugated α-bungarotoxin (α-BTX, 1∶500 Invitrogen) for 1 hour at room temperature to label AChRs. Cultures were rinsed in PBS and mounted in 50% glycerol/PBS mixture containing 0.03 mg/ml ρ-phenylenediamine.

**Table 1:** Primary Antibodies.

Antiserum	Host Species	Dilution	Clonality	Source
Dihydropyridine Receptor (α2 subunit, L-type VDCC)	Mouse	1∶500	Monoclonal	Sigma, Saint Louis, Missouri
GFP	Rabbit	1∶2000	Polyclonal	Chemicon, Temecula, CA
mAb 35 (non-blocking AChR)	Mouse	*1∶1000	Monoclonal	Developmental Studies Hybridoma Bank (DSHB), Iowa City, IA
NCAM (5e – chick specific)	Mouse	*1∶1000	Monoclonal	DSHB, Iowa City, IA
NCAM (CD56- rodent specific)	Mouse	1∶20001∶5000	Monoclonal	BD Biosciences, Franklin Lakes, NJ
SV2	Mouse	1∶50	Monoclonal	DSHB, Iowa City, IA
Synaptophysin	Rabbit	1∶500	Polyclonal	Zymed, San Fransisco, CA

* DSHB supernatant concentrated by filter centrifugation.

**Table 2:** Secondary Antibodies.

*Secondary antibody*	*Dilution*	*Conjugate*	*Source*
Goat anti-mouse IgG	1∶500	Alexa Fluor488	Invitrogen
Goat anti-mouse IgG	1∶500	Cy 3	Jackson Immunoresearch, Baltimore, PA
Goat anti-mouse IgG	1∶500	Alexa Fluor647	Invitrogen
Goat anti-rabbit IgG	1∶500	Alexa Fluor488	Invitrogen
Goat anti-rabbit IgG	1∶500	Cy 3	Jackson Immunoresearch, Baltimore, PA
Goat anti-rabbit IgG	1∶500	AlexaFluor647	Invitrogen
Goat anti-mouse IgG	1∶5000	HRP	Chemicon, Temecula, CA

### Quantification of endplate morphology

For quantification of postsynaptic endplate morphology, cultures were digitally photographed using a wide-field fluorescence microscope equipped with a broad focal plane lens (Leica Microsystems, Bannockburn, IL, USA) attached to a digital camera (C4742; Hamamatsu, Japan). Synapses were only quantified if the captured imaged accurately reflected the entire three-dimensional structure of the synapse. Captured images were analyzed for areas using IPLab (Version 4.0; BD Biosciences) or ImageJ software. All representative images are shown as collapsed z-stacks acquired using an LSM510 laser scanning confocal microscope (Zeiss Microimaging, Thornwood, NY, USA) and managed using Zen 2009 software (Zeiss Microimaging).

### Intracellular electrophysiology of co-cultured NMJs

Experiments were performed at room temperature in a recording chamber containing 1 ml of 50% Neurobasal/50% Hibernate low fluorescence solution (Brain Bits, Springfield, IL) supplemented with B27 (Sigma). Postsynaptic endplates were identified by the application of a non-blocking AChR antibody, mAb35 (Table 1) conjugated to an Alexa Fluor546 fluorchrome using a Monoclonal Antibody Labeling Kit (Invitrogen) for 1 hour prior to the recording session. NMJs were identified by the expression of GFP in apposition to mAb35 fluorescence identified using a CCD camera coupled to an Olympus upright fluorescence microscope (Centre Valley, PA). Images were captured using a Nikon digital camera. Micropipettes used for recordings had tip resistances between 10 and 50 MΩ and were filled with 3 M KCl. Reponses were recorded with a Sutter amplifier and processed with Clampex 10.2 software (Molecular Devices). All data were analyzed using MiniAnalysis (Synaptosoft, Decatur, GA). Quantal contents were determined by the direct method (*m* =  spontaneous endplate potential/miniature endplate potential; sEPP/mEPP) using the mean mEPP as determined following application of 2.5 μM TTX. In some cases, 5 μM μ-conotoxin GIIIB (Alomone Labs, Jerusalem, Israel) was added to the recording solution to block Na^+^ channel-mediated myotube contraction.

### FM4-64 loading

Co-cultures were incubated with 5 μM FM4-64FX and motor terminals were either loaded by spontaneous endocytosis in the presence of 2.5 μM TTX or electrically stimulated. For stimulation-induced FM4-64-uptake, cultures were preincubated with drug or vehicle for 30 minutes after which FM4-64 was added for 5 minutes before stimulation. Co-cultures were then stimulated with 1-second trains of 50 Hz stimuli, delivered at a 0.5 Hz train rate for 5 minutes. A maximum of 4 wells containing co-cultures were stimulated at a time with silver electrodes immersed into the culture media. Experiments always included simultaneous loading of 2 cultures from each genotype, one acting as a vehicle control and one as a drug treatment. Stimuli were 20 V pulses with 0.5 ms pulse widths. Cultures were then left to rest for 10 minutes following the stimulus train to allow for compensatory endocytosis [28], washed in media with reduced extracellular calcium concentration (1∶3 Neurobasal/HBSS solution) and with 2.5 μM TTX to inhibit further synaptic vesicle cycling then fixed with 3.7% formaldehyde. α-BTX was applied to the final wash cycle to label postsynaptic AChRs. Z stacks of identified NMJs were captured with an LSM510 laser scanning confocal microscope (Zeiss Microimaging, Thornwood, NY, USA) and managed using Zen 2009 software (Zeiss Microimaging). GFP fluorescence was excited with a 488 nm excitation laser and emission was captured with 500–530 nm bandpass filter. α-BTX fluorescence was excited with a 543 nm laser excitation and emission was captured with a 565–615 nm bandpass filter. FM4-64FX was excited with a 488 nm laser and emission was captured with a 685 longpass filter. FM4-64 fluorescence intensity was quantified using Zen software.

### Western blot

Cell lysates were collected by sonication in the presence of extraction buffer as described previously [29]. Briefly, co-cultures or ESMN embryoid bodies were lysed in extraction buffer containing; 50 mM Hepes, 150 mM NaCl, 1 mM EDTA, 2 mM PMSF, 100 μg/ml leupeptin, 0.2 TIU/ml aprotinin and 1% NP-4 in the presence of a cocktail of protease inhibitors (Complete Mini, Roche Diagnostics, Mannheim Germany). Samples were separated on a 6% acrylamide gel, then transferred onto an Immobilon-P membrane. Membranes were probed overnight with primary antibodies (Table 1) diluted in 1% milk TBS-T, then probed with secondary antibodies (Table 2) for 1 hour at room temperature. Membranes were developed on Kodak film (X-OMAT Blue Film XB, Kodak, Rochester, NY) using chemiluminescence (SuperSignal West Pico Chemiluminescent Substrate, Thermo Scientific, Rockford Il).

### Drug treatments

For all experiments including the use of drug treatments, drug solvents (vehicle; EtOH or DMSO) were used as controls. Nifedipine (Sigma) was dissolved in EtOH and used at 50 μM for acute experiments, while 5 μM was used for chronic experiments. ω-agatoxin IVA (Alomone Labs, Jerusalem, Israel) was dissolved in distilled water and used at a final concentration of 100 nM. Tetrodotoxin (TTX; Alomone Labs) was dissolved in distilled water and used at final concentrations between 2.5 and 5 μM. D-tubocurarine chloride hydrate (Sigma) was dissolved in distilled water and used at 50 μM. Dynasore (Sigma) was dissolved in DMSO and used at a final concentration of 90 μM.

### Data Acquisition and Statistical Analysis

An analyzer blinded to the experimental conditions performed all data analyses. Two tailed-Student's t-tests were used when comparing between two groups if normality was achieved. If normality was not achieved, Mann-Whitney tests were used to compare between groups. One-way ANOVA on ranks were performed to examine the differences between groups over time. Dunn's pairwise multiple comparisons test were then used to determine where significant differences occurred if the F-value exceeded F-critical. Statistical significance was considered to be achieved when *P*<0.05.

## Results

### Development of a stem cell-based culture system to study NMJs

To determine whether an *in vitro*, stem-cell based culture system can be used to study disorders of the NMJ we co-cultured wild-type (*NCAM^+/+^*) and *NCAM^-/-^* ESCMNs with chick myofibers. Because MNDs are not embryonic lethal, we chose to examine NMJs lacking a protein (i.e. NCAM) that when absent, reduces the size of the NMJ and alters the mode synaptic vesicle recycling, but does not affect NMJ formation [16], [17], [19], [20], [22].

**Figure 1:**
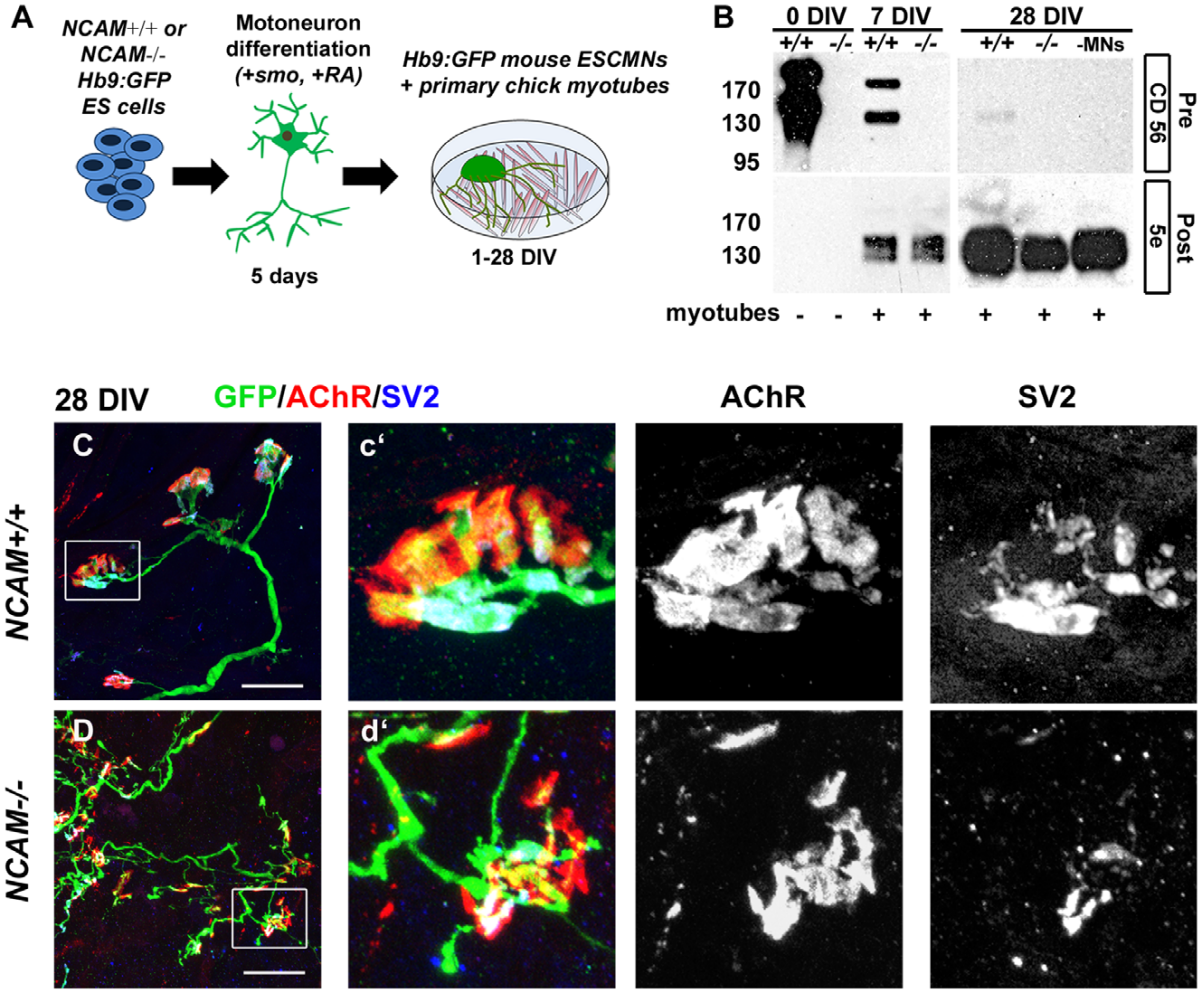
NCAM140 and 180 are expressed by ESCMNs and are necessary for mature NMJ formation in ESCMN/myofiber co-cultures. (**A**) Schematic illustration ESCMN differentiation and co-culture experiments. (**B**) Western blot analysis of Endo-N treated whole cell culture lysates obtained from cultures of *NCAM^+/+^* (+/+) or *NCAM^-/-^* (-/-) ESCMNs labeled with rodent-specific (CD56) or chick-specific (5e) NCAM antibodies in presence (+) or absence (-) of myotubes. (**C–D**) 28 DIV co-cultures labeled with α-BTX (AChR) and antibodies against GFP and SV2. Scale bars 20 μm. Boxed region is shown in c′ and d′.

Motor neurons were derived from mouse ES cells isolated from *NCAM^+/+^* and *NCAM^-/-^* mice expressing eGFP under the control of the Hb9 promoter. Hb9 is expressed by spinal motor neurons [27] and faithfully reports motor neuron identity *in vitro* [10], [11]. Consequently, eGFP expression was used throughout this study to identify fully differentiated motor neurons. ES cells were directed to differentiate into motor neurons using retinoic acid (+RA) and a smoothened agonist (Fig. 1A; +smo) [9]–[11] (reviewed by [30]). Embryoid bodies of aggregated *NCAM^+/+^* or *NCAM^-/-^* ESCMNs were plated onto a substrate of chick myotubes to allow for synapse formation (Fig 1A). The myotube cultures themselves were previously generated previously from myoblasts harvested from white leghorn chick external adductor muscles at stage 38. The anatomy and function of the NMJs were then studied one to 28 days later (Fig. 1A).

To verify the presence and absence of NCAM in *NCAM^+/+^* and *NCAM^-/-^* ESCMNs, respectively, we performed Western blot analysis on Endo-N pretreated protein homogenates collected from the differentiated ESCMNs prior to plating (Fig. 1B; 0 DIV), or from ESCM/myotube co-cultures 7 and 28 days after plating (Fig. 1B; 7 DIV, 28 DIV). Endo-N pre-treatment was used to remove polysialic acid from the NCAM protein in order to better identify individual NCAM isoforms [29]. Mouse- and chick-specific NCAM antibodies (CD56 and 5e, respectively) were used to detect NCAM expression by the motor neurons and muscle fibers, respectively (Fig 1B). NCAM 140 and 180 kD isoforms were both highly expressed by *NCAM^+/+^* ESCMNs in cell aggregates (i.e. 0 DIV) and when co-cultured with myotubes for 7 days (7 DIV). A low level of NCAM 140 was identified at 28 DIV (Fig 1. B). As expected, NCAM was absent from *NCAM^-/-^* ESCMNs at all time points (Fig. 1B). Chick myotubes, co-cultured with *NCAM^+/+^* or *NCAM^-/-^* ESCMNs for 7 (Fig 1B; 7 DIV), expressed comparable levels of NCAM 130 and 145 kD isoforms [29] indicating that the expression of postsynaptic NCAM was similar in the two conditions. Similar results were obtained when myotubes were assessed for NCAM expression in cultures grown with *NCAM^+/+^* ESCMNs, *NCAM^-/-^* ESCMNs, or without ESCMNs (-MNs) at 28 DIV (Fig. 1B; 28 DIV).

**Figure 2:**
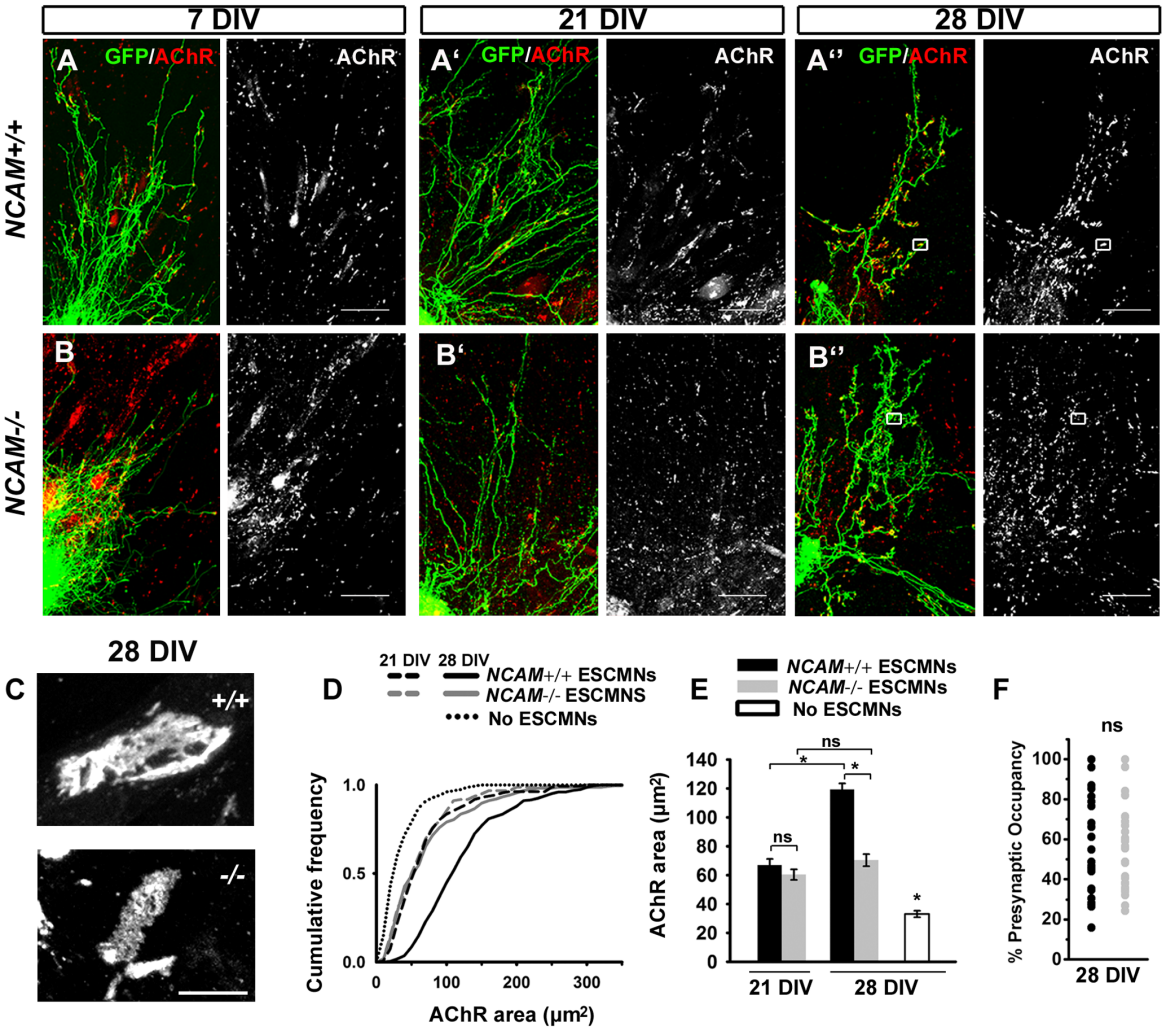
Presynaptic NCAM influences the maturation of NMJs formed by ESCMNs *in vitro*. (**A**) Representative images of co-cultures at 7, (**A′**) 21 and (**A″**) 28 DIV formed by *NCAM^+/+^* and (**B–B″**) *NCAM^-/-^* ESMNs. Scale bars 200 μm. (**C**) Magnification of boxed regions in A″ and B″ shows that NCAM*^+/+^* endplates are larger and more complex than those formed by *NCAM^-/-^* ESCMNs. Scale bar for both images 10 μm. (**D**) Cumulative frequency plot of AChR areas measured at 21 DIV and 28 DIV. Cultures of myotubes without ESCMNs were grown for 28 days as controls. (**E**) Mean ±SEM AChR areas measured in *NCAM^+/+^* and *NCAM^-/-^* cultures at DIV 21 and DIV 28. N = 158 synapses from 3 *NCAM^+/+^* cultures at 21 DIV; N = 158 synapses from 3 *NCAM^-/-^* cultures at 21 DIV; N = 225 synapses from 3 *NCAM^+/+^* cultures at 28 DIV; N = 225 synapses from 3 *NCAM^-/-^* cultures at 28 DIV; N = 152 endplates from 28 DIV myotube cultures without ESCMNs. * *P*<0.001 ANOVA on ranks and Dunn's pairwise multiple comparisons test. (F) The fractional occupancy (% occupancy) of endplates in *NCAM^+/+^* and *NCAM^-/-^* co-cultures does not differ at 28 DIV. Presynaptic areas were defined by SV2 immunofluorescence. *P* = 0.998 t-test.

**Figure 3:**
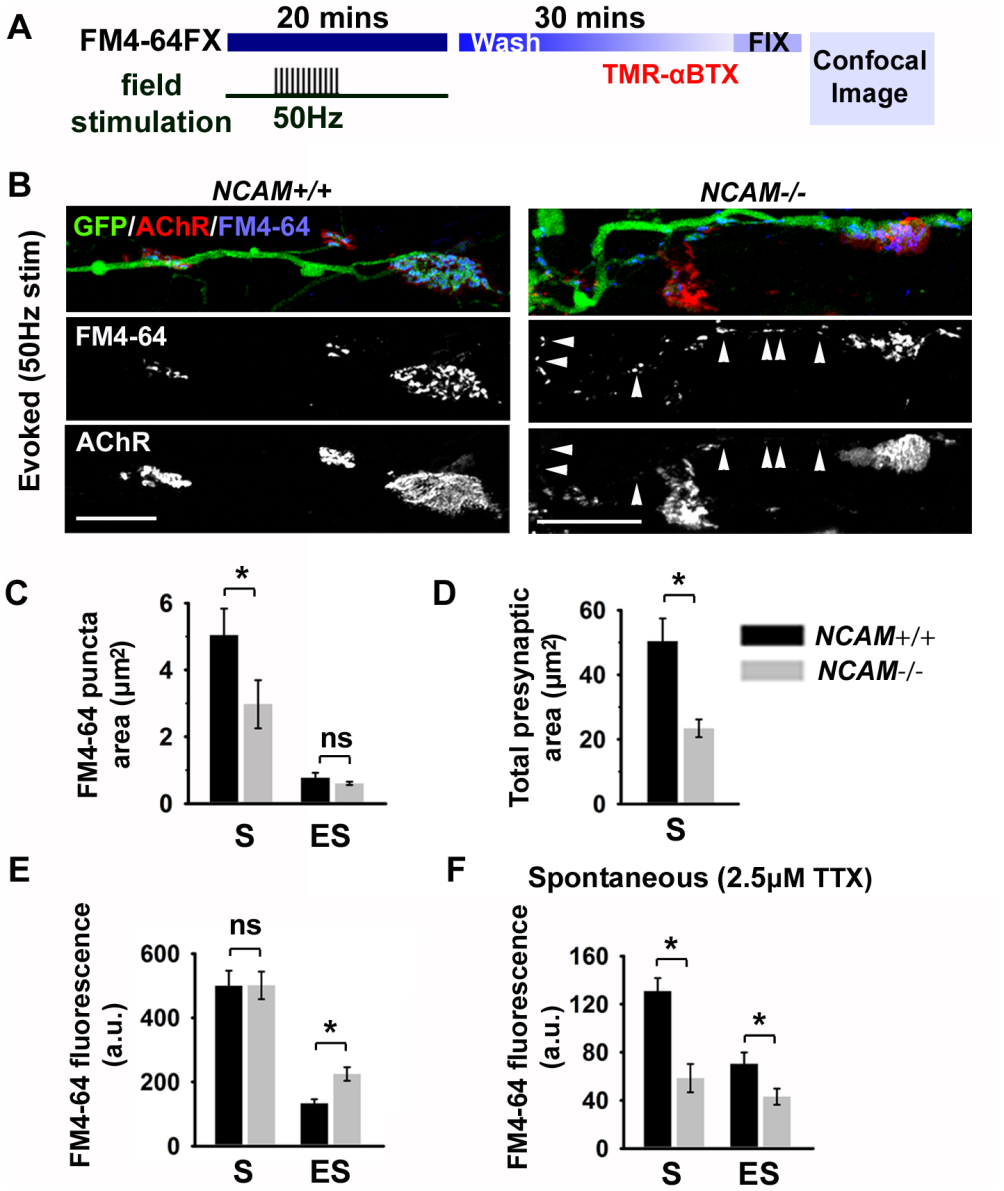
Presynaptic NCAM contributes to the functional differentiation of motor terminals *in vitro*. (**A**) Schematic illustrating FM4-64FX loading and imaging paradigm using repetitive 50 Hz field stimulation. (**B**) Representative images of FM4-64FX loading in the presynaptic terminal of *NCAM^+/+^* and *NCAM^-/-^* NMJs at 28 DIV. Arrows identify extra-synaptic FM4-64 puncta in an *NCAM^-/-^* ESCMN. Scale bars 20 μm. (**C**) Mean ±SEM area of individual FM4-64+ puncta at synaptic (S; defined as GFP^+^ and TMR-BTX^+^ regions) and extra-synaptic (ES; defined as GFP^+^ and TMR-BTX^-^ regions). *P = 0.05 ANOVA on ranks and Dunn's multiple comparisons test. (**D**) Mean ±SEM total presynaptic area (defined as the sum of all synaptic FM4-64 puncta overlying a AChR^+^ endplate). *P*<0.001 Mann Whitney rank sum test. (**E**) Mean ±SEM evoked FM4-64 fluorescence at synaptic (S) and extra-synaptic (ES) sites. N = 37 synapses from 9 *NCAM^+/+^* cultures; N = 61 from 11 *NCAM^-/-^* cultures. *P* = 0.831 Mann Whitney rank sum test at synaptic sites. *P* = 0.004 Mann Whitney rank sum test at extra-synaptic sites. (**F**) Mean ±SEM spontaneous FM4-64 uptake in the presence of 2.5 μM TTX at synaptic (S) and extra-synaptic (ES) sites. Extra-synaptic sites were measured within 50 μm of each synaptic region. *P*<0.001 Mann Whitney rank sum test at synaptic sites; *P* = 0.029 t-test at extra-synaptic sites. N = 32 synapses from 6 *NCAM^+/+^* cultures; N = 28 synapses from 6 *NCAM^-/-^* cultures.

When *NCAM^+/+^* ESCMNs were grown with muscle fibers for 28 days they formed NMJs that exhibited mature morphology and resembled NMJs of postnatal mice (Fig. 1C, c′). Clusters of postsynaptic acetylcholine receptors (AChRs) formed large and complex structures, and were closely apposed by synaptic vesicles (Fig 1. C, c′; revealed by SV2 immunolabeling) that had accumulated at the distal ends of GFP+ motor axons. *NCAM^-/-^* ESCMNs formed NMJs that were strikingly less mature and more disorganized from those formed by *NCAM^+/+^* ESCMNs (Fig. 1D, d′). In particular, postsynaptic endplates were smaller (also see Fig. 2), and presynaptic motor terminals were disorganized and exhibited reduced synaptic vesicle clustering (also see Fig. 3). These findings demonstrate that the ESCMN/myotubes co-culture system is an effective means to model synapse formation *in vitro*.

### Maturation of the NMJ is compromised in co-cultures containing *NCAM^-/-^* ESCMNs

Motor neurons form smaller NMJs in *NCAM^-/-^* mice compared to aged matched control animals [16], [17]. To further examine whether endplate formation is similarly compromised *in vitro* when NCAM is absent, we co-cultured *NCAM^+/+^* or *NCAM^-/-^* ESCMNs with chick myotubes for 7-28 DIV. Figure 2 shows that neurite outgrowth was not visibly different between the two genotypes after 7, 21 and 28 DIV (Fig. 2A-B). However, as occurs in *NCAM^-/-^* mice [16], the endplates were significantly smaller in co-cultures containing *NCAM^-/-^* ESCMNs after 28 DIV (Fig. 2C-E). This difference was due to lack of endplate growth after 21 DIV rather than delayed formation, as endplates formed by both genotypes were the same size after 21 DIV (Fig. 2D,E). Moreover, the fractional occupancy of postsynaptic endplates by presynaptic terminals was determined by comparing the area occupied by SV2 immunofluorescence to the area occupied by AChRs at 25 NMJs of each genotype at 28 DIV (Fig. 2F). Although highly variable, the fractional occupancy of presynaptic terminals did not differ between genotypes (Fig. 2F; *P* = 0.998, two-tailed t-test). Moreover, this analysis revealed that presynaptic areas scaled linearly with postsynaptic areas at NMJs of both genotypes (*NCAM^+/+^*, Pearson's r value  = 0.669; *P*<0.001; *NCAM^-/-^*, Pearson's r value  = 0.885; *P*<0.001), and SV2-labeled *NCAM^-/-^* motor terminals were significantly smaller than *NCAM^+/+^* motor terminals (28.77 μm^2^±5.03 vs. 60.77 μm^2^±6.96 *NCAM^-/-^* vs. *NCAM^+/+^*, *P* = 0.00128, two-tailed t-test). In addition, while not quantified, the endplates in the *NCAM^+/+^* ESMN co-cultures were more complex compared to those in the *NCAM^-/-^* cultures (Fig. 2C). Taken together, these findings show that ESCMNs can form stable NMJs *in vitro* that continue to mature for at least 4 weeks. Furthermore, as occurs *in vivo*, this maturation process is compromised when NCAM is absent.

### Synaptic vesicle cycling is abnormal in *NCAM^-/-^* ESCMN/chick myotube co-cultures

Next, we quantitatively examined presynaptic differentiation in ESCMN/myotubes co-cultures using FM4-64 as a marker of active presynaptic terminals. Previous studies on *NCAM^-/-^* mice showed that presynaptic differentiation is altered at NMJs when NCAM is absent [16], [20]. For example, Landmesser and colleagues [20] used FM styryl dyes to show that synaptic vesicles are abnormally cycled along the axon in *NCAM^-/-^* mice rather than being released solely at the NMJ. To investigate whether *NCAM^-/-^* ESCMNs exhibit a similar phenotype we repetitively stimulated 28 day old co-cultures with 1-second trains of electrical pulses, every two seconds, for 5 minutes in the presence of FM4-64 to load the dye into recycling synaptic vesicles (Fig. 3A). The cultures were then washed for 30 minutes and incubated with α-BTX to visualize postsynaptic AChRs (Fig. 3A–B). This stimulation paradigm effectively loaded cycled vesicles with FM4-64 at the presynaptic terminals in the *NCAM^+/+^* and *NCAM^-/-^* co-cultures (Fig. 3B). However, there were several notable differences between the two genotypes in the organization of the cycled FM4-64^+^ membranes. First, unlike the *NCAM^+/+^* co-cultures, numerous FM4-64 puncta were located at extra-synaptic regions along the neurites of *NCAM^-/-^* ESCMNs (Fig 3B; arrowheads). Second, consistent with Fig. 2F, the sizes of individual FM4-64 puncta at synaptic regions (Fig. 3C; S - defined as regions of overlap between GFP^+^ and TMR-BTX^+^ fluorescence) were smaller in *NCAM^-/-^* cultures, although the extra-synaptic puncta (ES - defined as GFP^+^ and TMR-BTX^-^ regions) were similar in size (Fig. 3C, ES). Third, the total presynaptic area (the aggregates of individual S puncta) was smaller in ESCMN *NCAM^-/-^* co-cultures (Fig. 3D). Moreover, although *NCAM^-/-^* motor terminals are substantially smaller than *NCAM^+/+^*, the intensity of FM4-64 fluorescence per unit area was similar between genotypes at the synapse (Fig 3E; S). Interestingly, *NCAM^-/-^* ESCMNs contained significantly higher levels of FM dye at extra-synaptic regions (Fig. 3E; ES), suggesting that functional synaptic vesicles are displaced in the absence of presynaptic NCAM. These observations were confirmed in cultures where synaptic vesicle recycling was stimulated using 70 mM KCl (data not shown). Finally, less FM dye was endocytosed in the presence of TTX at synaptic and extra-synaptic sites in *NCAM^-/-^* ESCMNs (Fig 3F), demonstrating that spontaneous recycling is also altered by the absence of presynaptic NCAM. These results indicate that active synaptic vesicle cycling, which only takes place at the synapses of healthy adult NMJs [31], is abnormal in *NCAM^-/-^* ESCMN/myotube co-cultures.

### Cultured *NCAM^-/-^* ESCMNs have compromised neurotransmission at the NMJ

**Figure 4:**
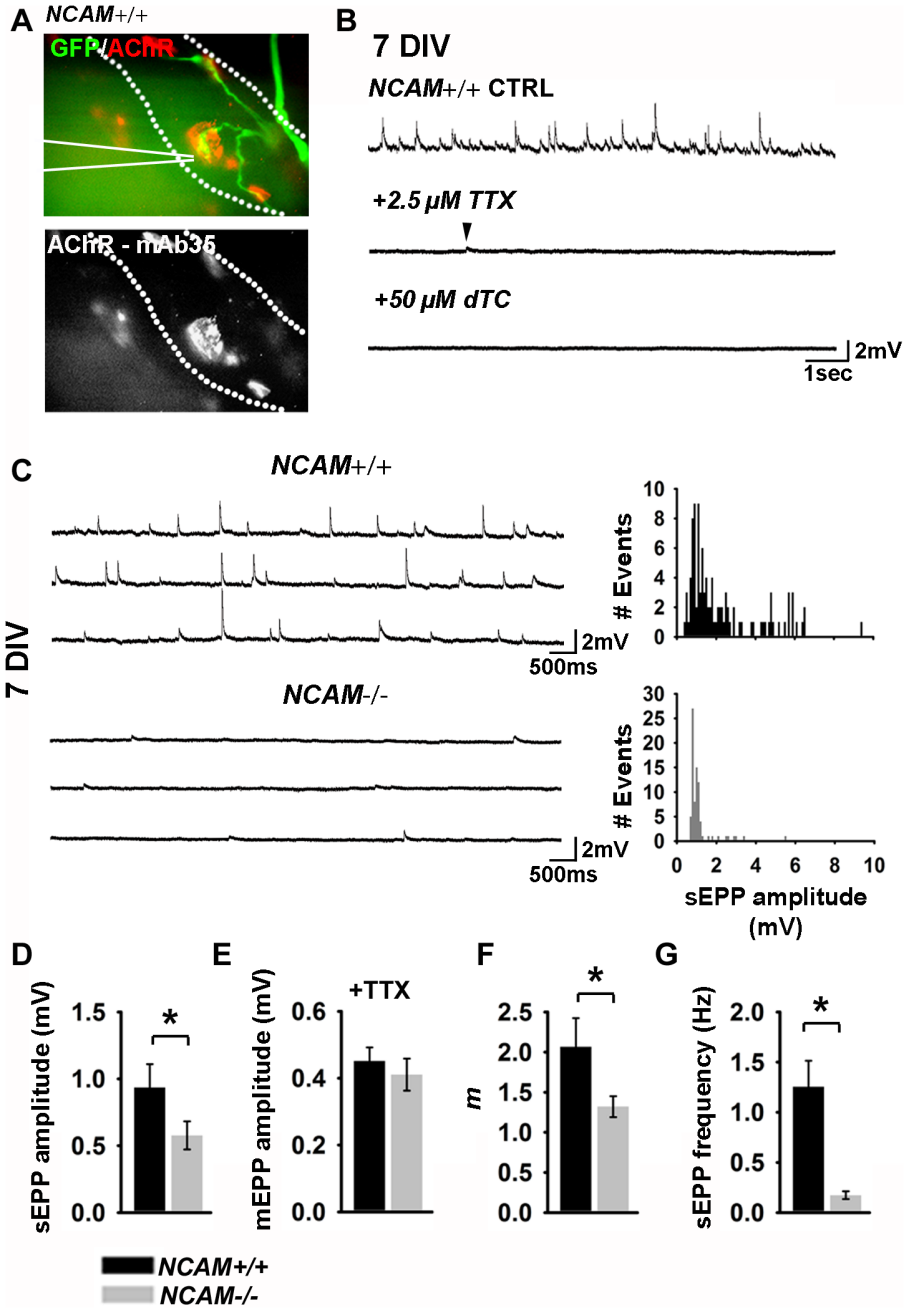
Presynaptic NCAM regulates the strength of neurotransmission at newly formed NMJs *in vitro*. (**A**) Representative image of a typical NMJ targeted for intracellular recording at 7 DIV. GFP fluorescence was imaged to identify presynaptic terminals and postsynaptic endplates were identified using mAB35 conjugated to Alexa546. Dashed outline indicates the postsynaptic muscle fiber; solid white lines indicate the position of the intracellular recording electrode. (**B**) Representative recording traces from an *NCAM^+/+^* NMJ in normal recording media (top), after addition of 2.5 μm tetrodotoxin (TTX; middle) and following addition of 50 μm tubocurarine (dTC; bottom). Arrowhead indicates the presence of a spontaneous miniature endplate potential (mEPP) that persist following the addition of TTX. (**C**) Representative recording traces obtained in control media (left) and frequency histograms (right) of synaptic events at *NCAM^+/+^* and *NCAM^-/-^* NMJs at 7 DIV. (**D**) Mean ±SEM spontaneous endplate potential (sEPP) amplitude. *P* = 0.013 Mann-Whitney rank sum test. (**E**) Mean ±SEM of mEPPs recorded in the presence of 2.5 μM TTX. (**F**) Mean ±SEM quantal content (m) obtained by the ratio of sEPP amplitude/mean mEPP amplitude. *P* = 0.008 Mann Whitney rank sum test. (**G**) Mean ±SEM sEPP frequency recorded in normal media. *P* = 0.002 Mann Whitney rank sum test. N = 17 cells from 6 *NCAM^+/+^* cultures; 18 cells from 7 *NCAM^-/-^* cultures.

Previous studies have shown that neurotransmission at the NMJ is compromised when NCAM is absent in adult mice [16], [20], [21]. Furthermore, spontaneous miniature endplate potentials (mEPPs) occur less frequently and the amplitudes of evoked endplate potentials (EPPs) were significantly smaller than normal at reinnervated endplates in *NCAM^-/-^* mice [22]. Quantal content at reinnervated endplates lacking NCAM was also less than normal because the amplitudes of the mEPPs were unchanged [22]. To examine whether ESMN/myotube cultures lacking presynaptic NCAM exhibit similar neurotransmission abnormalities, we cultured *NCAM^+/+^* or *NCAM^-/-^* ESCMNs on chick myotubes and recorded mEPPs and spontaneously occurring EPPs (sEPPs) at functional NMJs after 7 DIV. Functional NMJs were identified as clusters of AChRs that were contacted by a single eGFP^+^ neurite (Fig. 4A). AChRs were visualized in live cultures using a rhodamine-conjugated antibody that binds to AChRs but does not block their function [20]. Consistent with previous findings [10], sEPPs with varying amplitudes were recorded from NMJs in *NCAM^+/+^* ESCMN/myotube co-cultures (Fig. 4B). The sEPPs were largely action-potential driven, as application of 2.5 μM tetrodotoxin (TTX) dramatically reduced their frequency and abolished the largest events. The remaining EPPs in the TTX treated cultures were mEPPs (Fig. 4B; arrow) because mEPPs are, by definition, spontaneously evoked events that are not sensitive to TTX. As expected, application of d-tubocurarine (dTC; 50 μM) completely abolished all events in the co-cultures indicating that the EPPs were evoked by cholinergic neurotransmission (Fig 4B).

The sEPPs recorded from *NCAM^-/-^* NMJs were significantly smaller (Fig. 4C–D), and less frequent (Fig. 4C, G), than those recorded from *NCAM^+/+^* junctions after 7 DIV. However, this analysis does not distinguish between reduced sEPP frequencies due to altered action potential initiation and/or propagation from reduced sEPP frequencies due to a low probability of release in response action potential infiltration of the motor terminal. We therefore measured sEPP amplitudes in the presence and absence of action potentials (i.e. mEPPs). The amplitude of the spontaneous mEPPs, recorded in the presence of TTX, did not differ between phenotypes (Fig. 4E) suggesting that quantal content (*m*), and not the density of post-synaptic AChRs, was reduced at *NCAM^-/-^* NMJs. Indeed, when calculated using the direct method of quantal analysis (i.e. dividing the amplitude of the sEPPs by the amplitude of the mEPPs recorded in the presence of TTX) quantal content (*m*) was found to be significantly lower at *NCAM^-/-^* NMJs (Fig. 4F). Moreover, treatment of *NCAM^+/+^* and *NCAM^-/-^* co-cultures with agatoxin (100 nM) reduced sEPP frequency in both cultures (data not shown), suggesting that spontaneous activity is driven, at least in part, by calcium influx through P/Q type calcium channels regardless of the presence of presynaptic NCAM. Spontaneous EPP amplitude did not differ between genotypes in the presence of ATX (data not shown). Together, these findings indicate that *NCAM^-/-^* ESCMN/myotube co-cultures exhibit functional properties similar to their endogenous counterparts when NCAM is absent presynaptically.

### Presynaptic NCAM co-localizes with proteins associated with synaptic vesicle cycling

**Figure 5:**
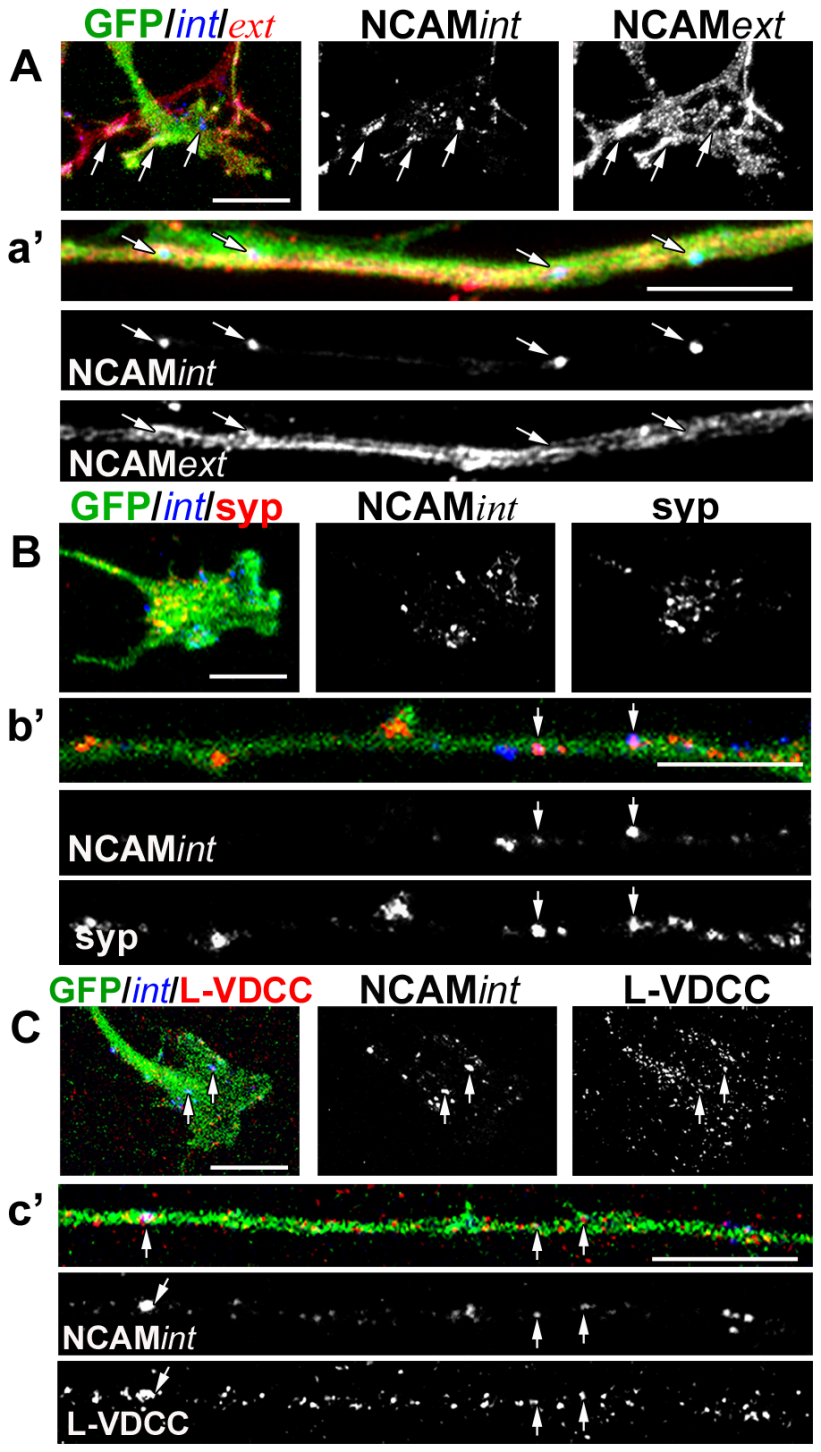
Endocytosed NCAM partially co-localizes with synaptic vesicles and L-type Ca^2+^ channels in growing ESCMN axons. (**A**) Growth cones and (a′) shaft of growing ESCMN axons at 1 DIV co-immunolabeled for NCAM*int* and NCAM*ext*. While NCAM*ext* is uniformly distributed along the cone and axonal shaft, NCAM*int* is distributed in discrete puncta (arrows). (**B, b′**) Co-immunofluorescence demonstrates partial co-distribution of NCAM*int* with synaptophysin (syp). The co-localization coefficient of NCAM*int* with syp in a single optical plane of the growth cone shown *B* is 0.077, and for the axon shown in *b′* is 0.689. (**C, c′**) Co-immunofluorescence demonstrates partial co-distribution NCAM*int* and the L-type voltage dependent calcium channels (L-VDCCs). The co-localization coefficient of NCAM*int* with L-VDCCs in a single optical plane of the growth cone shown *C* is 0.450, and for the axon shown in *c′* is 0.124. Arrows denote co-distributed puncta. In *B* and *C* NCAM*ext* was labeled with Alexa350, but was not imaged. All scale bars 10 μm.

The functional deficits observed at NMJs formed by *NCAM^-/-^* ESCMNs, along with the abnormal distribution of synaptic vesicles along the axon shaft and observations of NCAM internalization in growth cones [32] prompted us to examine whether internalized NCAM is associated with structures involved in neurotransmission. To do so, we used a sequential immunostaining protocol to selectively label extracellular NCAM on the surface of ESCMNs (NCAM*ext*) as well as extracellular NCAM that had been internalized during the labeling period (NCAM*int*). In brief, *NCAM^+/+^* eGFP^+^ ESCMNs were grown in culture for one day and immunolabeled live with CD56 for one hour in presence of TTX. Cells were then fixed and labeled with a Cy3 conjugated secondary antibody in the absence of detergent to label only surface antigens. The cells were then permeabilized with 0.3% TritonX and incubated with an Alexa Fluor647 secondary antibody. This sequential NCAM immunostaining protocol selectively labels NCAM*ext* with Cy3 and NCAM*int* with Alexa Fluor647 (Fig. 5A). Figure 5 shows a typical example of a growth cone (Fig. 5A) and neurite shaft (Fig. 5a′) immunolabeled for NCAM*ext* and NCAM*int*. Note that while extracellular NCAM is highly expressed throughout the growth cone and shaft, intracellular NCAM is localized to discrete regions (Fig. 5a′; arrowhead). Using the same sequential immunostaining technique, we co-immunolabeled *NCAM^+/+^* ESCMNs after 1 DIV with additional antibodies against two synapse-associated proteins (Fig. 5B–C). Internalized NCAM was found to associate with syp+ synaptic vesicles, although this association was comparatively more robust along the length of the axon (Fig. 5b′, arrows) than at the growth cone (Fig. 5B). This observation prompted us to examine whether NCAM*int* associates with L-VDCCs, as vesicle recycling along the shaft of motor neuron axons is sensitive to L-VDCC inhibition [26], and this immature form of vesicle recycling is abnormally preserved at NMJs *in vivo* in the absence of NCAM [20]. Indeed, we observed several NCAM*int* puncta which co-localized with L-VDCC immunohistochemistry in the growth cone (Fig. 5C), and along the axon shaft (Fig. 5c′). Because we immunolabeled surface NCAM in live cultures, these findings suggest that NCAM is endocytosed in growing neurites and becomes associated with several presynaptic-related proteins.

### NCAM is endocytosed at synaptic regions in vitro

**Figure 6:**
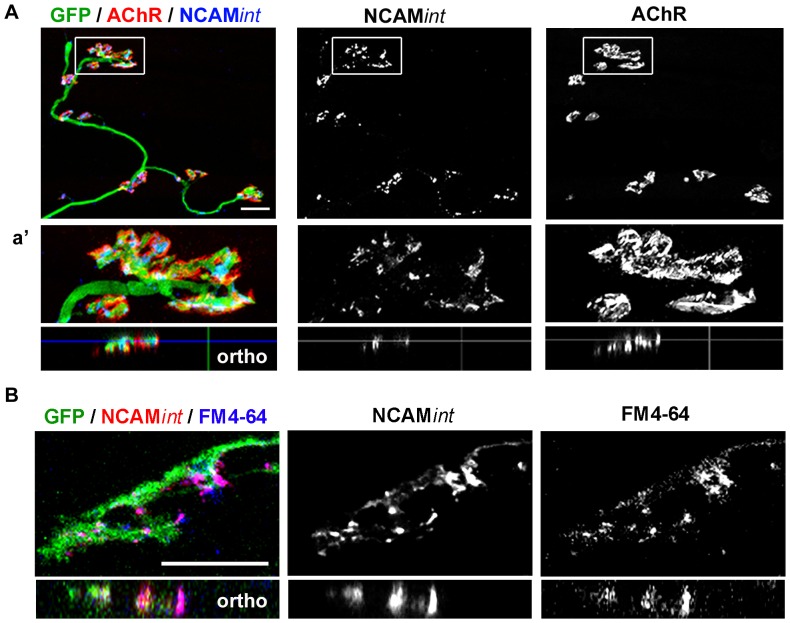
NCAM is endocytosed at synaptic regions *in vitro*. (**A**) Representative images of *NCAM^+/+^* DIV 28 co-culture immunolabeled for NCAM*int* and GFP and labeled with α-BTX to identify postsynaptic AChRs. NCAM*ext* was labeled with Alexa350 and was not imaged. (a′) Magnification of boxed region in *A* demonstrating punctate pattern of NCAM*int* distribution in the motor terminal. Orthogonal images are shown below and confirm a presynaptic localization of NCAM*int*. (**B**) Representative image of *NCAM^+/+^* DIV 28 motor terminal which had been live immunolabeled with NCAM*int* and FM4-64FX in the presence of 2.5 μM TTX for 30 minutes. Arrows demonstrate co-localization of NCAM*int* and FM4-64 signals. All scale bars 10 μm.

Next, we examined whether NCAM is similarly recycled at mature synapses formed by ESCMNs (Fig. 6). We performed the same sequential immunolabeling technique to image NCAM*int* in mature (i.e. 28 DIV) *NCAM^+/+^*/myotube co-cultures (Fig. 6A). Endocytosed NCAM localized nearly exclusively to synaptic sites, where it appeared in a punctate pattern throughout the motor terminal (Fig. 6a′). Orthogonal views (ortho) confirm presynaptic localization of NCAM*int* (Fig. 6a′). We further confirmed that NCAMint reflects the presence of endocytosed membrane containing NCAM by pairing the NCAM*int* immunostaining approach with FM4-64 labeling (Fig. 6B). Indeed, the NCAM*int* and FM4-64 signals closely overlapped (Fig. 6B), confirming that NCAM endocytosis may reflect at least a portion of synaptic vesicle endocytosis in mature co-cultures.

### NCAM regulates endocytosis

**Figure 7:**
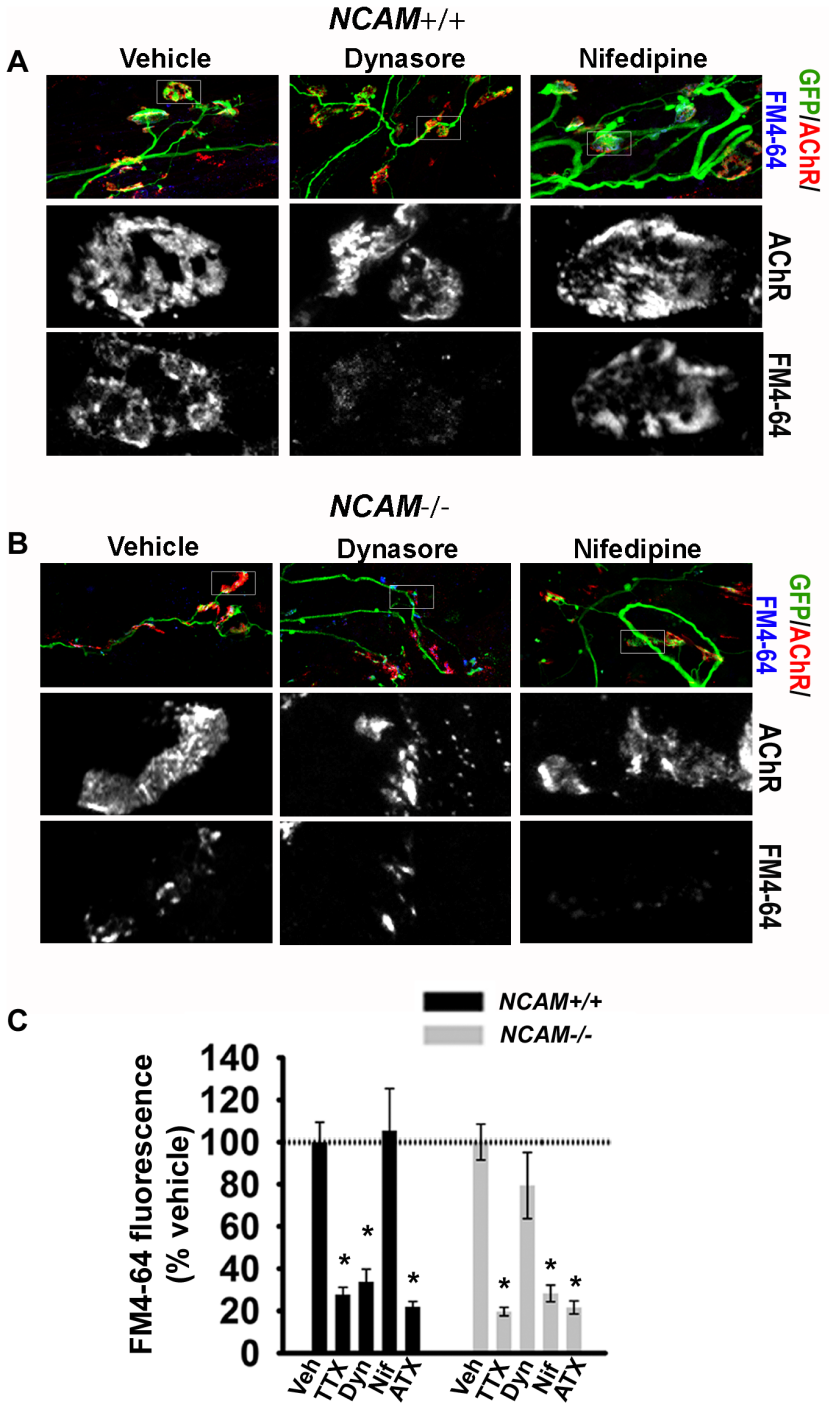
Endocytosis at *NCAM-/-* motor terminals is sensitive to inhibition by nifedipine, but insensitive to inhibition by dynasore. (**A**) Representative images of NMJs formed by *NCAM^+/+^* and (**B**) *NCAM^-/-^* ESMNs loaded with FM4-64 by 50 Hz stimulation in the presence of nifedipine (50 μM), dynasore (90 μM) or vehicle (DMSO). (**C**) Mean ±SEM FM4-64 fluorescence measured in cultures incubated with vehicle (veh), tetrodotoxin (TTX), dynasore (Dyn), nifedipine (Nif), and agatoxin (ATX). Each value was normalized to the mean vehicle value of each genotype. *P<0.001 one-way ANOVA on ranks and Dunn's pairwise multiple comparisons test, performed independently for each genotype. Vehicle: N = 37 synapses from 9 *NCAM^+/+^* cultures; N = 61 synapses from 11 *NCAM^-/-^* cultures. TTX: N = 15 synapses from 6 *NCAM^+/+^* cultures; N = 19 synapses from 4 *NCAM^-/-^* cultures. Dyn: N = 19 synapses from 6 *NCAM^+/+^* cultures; N = 21 synapses from 5 *NCAM^-/-^* cultures. Nif: N = 14 synapses from 3 *NCAM^+/+^* cultures; N = 33 synapses from 5 *NCAM^-/-^* cultures. ATX: N = 11 synapses from 3 *NCAM^+/+^* cultures; N = 14 synapses from 3 *NCAM^-/-^* cultures.

Synaptic vesicle cycling at the NMJ transitions from an L-VDCC-dependent to an L-VDCC-independent form of endocytosis [26], [33]. This transition is regulated *in vivo*, at least in part, by NCAM [20]. To examine whether cultured *NCAM^+/+^* ESCMNs develop “mature” synaptic vesicle dynamics that are independent of L-VDCC we loaded 28 DIV *NCAM^+/+^* ESCMNs co-cultures with FM4-64, in the presence or absence of synaptic vesicle cycle inhibitors, using the same stimulation paradigm described above (Fig. 7A,C). To assess the role of presynaptic NCAM in this maturation process we performed a second set of experiments using *NCAM^-/-^* ESCMN co-cultures (Fig. 7B,C). As expected, TTX inhibited activity-dependent endocytosis in both *NCAM^+/+^* and *NCAM^-/-^* co-cultures, as did the P/Q-type Ca^2+^ channel blocker agatoxin (ATX; 100 nm) (Fig 7C). Inhibiting dynamin, a GTPase essential to endocytic function, with dynasore (90 μM) [34], completely blocked activity-dependent endocytosis in *NCAM^+/+^*, but not *NCAM^-/-^* cultures (Fig 7A-C). Conversely, while nifedipine (50 μM), an L-VDCC antagonist, had no effect on endocytosis at *NCAM^+/+^* NMJs, it completely blocked endocytosis at *NCAM^-/-^* NMJs (Fig 7A-C). Taken together, these results confirm observations from previous *in vivo* [19], [20], and recent *in vitro* studies [26], [35] showing that NCAM regulates the transition from an immature (L-VDCC-dependent) to a mature (L-VDCC-independent) form of synaptic vesicle endocytosis. Furthermore, they show that NMJs formed by ESCMNs respond to pharmacological interventions in a manner that is analogous to their endogenous counterparts.

### Reversal of synaptic deficits with chronic inhibition of L-VDCC activity in *NCAM^-/-^* ESCMNs

**Figure 8:**
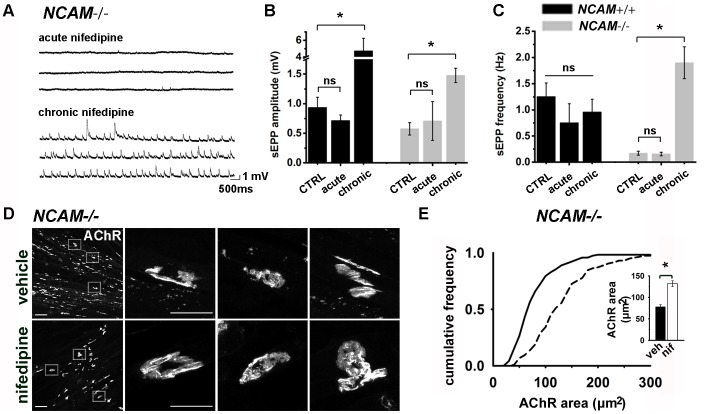
Chronic treatment of *NCAM^-/-^* cultures with nifedipine improves neurotransmission and enhances synaptogenesis. (**A**) Representative sharp electrode intracellular recording traces from 7 DIV *NCAM^-/-^* NMJs treated with nifedipine for 10 minutes (acute nifedipine, 50 μM; top) or once a day for 5 days (chronic nifedipine, 5 μM; bottom). (**B**) Mean ±SEM sEPP amplitude following acute or chronic treatment with nifedipine. *P<0.001 ANOVA on ranks and Dunn's pairwise multiple comparisons test. (**C**) Mean ±SEM sEPP frequency following acute or chronic treatment with nifedipine. * *P*<0.001 ANOVA on ranks and Dunn's pairwise multiple comparisons test. CTRL: N = 28 synapses from 9 *NCAM^-/-^* cultures, N = 17 synapses from 6 *NCAM^+/+^* cultures. Acute: N = 4 synapses from 4 *NCAM^-/-^* cultures, N = 4 synapses from 4 *NCAM^+/+^* cultures. Chronic N = 17 synapses from 5 *NCAM^-/-^* cultures, N = 14 synapses from 3 *NCAM^+/+^* cultures. (**D**) Representative images of 28 DIV *NCAM^-/-^* cultures labeled with α-BTX following 7 day treatment with vehicle (DMSO) or nifedpine (5 μM). Scale bar 50 μm low magnification; 20 μm high magnification. (**E**) Cumulative frequency plot and (*insert*) mean ±SEM of AChR areas measured from 28 DIV *NCAM^-/-^* cultures treated with vehicle or nifedipine (5 μM) daily for 7 days. * *P*<0.001 Mann Whitney rank sum test. N = 117 synapses from 4 *NCAM^-/-^* cultures for both conditions.

Although it is not known why NMJs switch from an L-VDCC-dependent to an L-VDCC-independent mechanism as they mature, we speculated that it occurs in order to maximize the efficiency of neurotransmission. To investigate this possibility, we inhibited L-VDCCs in *NCAM^+/+^* and *NCAM^-/-^* co-cultures with nifedipine either daily from 2 DIV to 7 DIV (i.e. chronic treatment), or applied nifedipine 10 minutes before recording sEPPs (i.e. acute treatment) (Fig. 8A). Figure 8A-C shows that, although acute treatment had little effect on the amplitude and frequency of sEPPs, chronic treatment significantly enhanced the amplitude of sEPPs in cultures of both genotypes and increased the frequency of the sEPPs in *NCAM^-/-^* co-cultures (Fig 8B–C). Therefore, consistent with *ex vivo* studies [33], inhibition of the L-VDCC-dependent recycling pathway significantly potentiates neurotransmission at newly formed NMJs *in vitro*.

Secondly, we speculated that abnormally prolonged presynaptic L-VDCC activity could alter the morphology of NMJs, as was observed in *NCAM^-/-^* ESCMN co-cultures. To test this, we chronically inhibited L-VDCCs with nifedipine in *NCAM^+/+^* and *NCAM^-/-^* ESCMN/myotube co-cultures daily for one week, starting at 21 DIV and examined NMJ morphology. We chose to start drug treatments after 21 DIV because the endplates in *NCAM^-/-^* ESCMN/myotube co-cultures were similar in size prior to that time point (Fig. 2D,E). NMJ maturation was quantified at 28 DIV by measuring endplate size. We found that chronic treatment of the *NCAM^-/-^* ESCMN/myotube co-cultures nearly doubled the size of the endplates (Fig 8D–E). Taken together, these results indicate that the transition in the mechanics of synaptic vesicle cycling, from an L-VDCC dependent form to an L-VDCC independent form, requires presynaptic NCAM and contributes to the maturation of newly formed synapses. Furthermore, they show that the ESCMN/chick myotube co-culture system is amenable to pharmacological manipulations designed to improve function at dysfunctional NMJs.

## Discussion

The present study shows that *NCAM^+/+^* and *NCAM^-/-^* ESCMNs form NMJs with co-cultured myotubes that are remarkably similar to their endogenous counterparts in wild-type and *NCAM^-/-^* mice, respectively. Compared to *NCAM^+/+^* ESCMNs, NMJs formed by *NCAM^-/-^* ESCMNs had limited growth, reduced neurotransmission and abnormal synaptic vesicle dynamics. Like NMJs in *NCAM^-/-^* mice, NMJs formed by *NCAM^-/-^* ESCMNs retained an immature mechanism of synaptic vesicle retrieval that remained L-VDCC sensitive and functioned independently of dynamin. The persistence of an L-VDCC sensitive mechanism for neurotransmission prompted us to examine whether chronic treatment of *NCAM^-/-^* ESCMN co-cultures with an L-VDCC antagonist would improve neurotransmission and synaptic growth. We found that neurotransmission and endplate morphologies reached near normal values in *NCAM^-/-^* ESCMN co-cultures treated with physiologically relevant concentrations of nifedipine. However, the present study cannot rule out a role of ESCMN firing activity, and a possible role of L-VDCC activity in the regulation of ESCMN excitability, on the development of NMJs in our co-culture system. We anticipate that future studies will begin to address these important issues. Taken together, we argue that the ESCMN/myotube co-culture system is a novel and useful bioassay for examining molecules regulating synaptic function and for screening therapeutics aimed at improving function of compromised NMJs in MNDs.

### The evolution of ESCMN-myotube co-cultures to study NMJ development and function

Co-cultures composed of primary spinal neurons and myotubes have been used over the past 40 years to study early events in synapse formation [36]–[40]. This simple technique was advanced over the past ten years when nerve-muscle co-cultures were developed using motor neurons derived from mouse or human pluripotent stem cells [10], [12], [13], [41], [42]. Regardless of the source (mouse or human; ES cell or iPS cell-derived), stem cell-derived motor neurons make functional connections with myotubes after 5-7 DIV [10], [11], [13]. However, few studies have examined NMJs in nerve-muscle co-cultures beyond 7 DIV. As a result, most studies report NMJs as immature post-synaptic plaque-like structures [10], [12], [13], [41], [42]. While these structures are very reminiscent of immature endplates in neonatal mice, they do not resemble mature endplates found in adult mice that appear as large pretzel-like structures.

Postnatal endplate maturation is characterized by the appearance of perforations in the plaque-like AChR reach structures, likely induced by concentrated regions of postsynaptic endocytosis [43]. We found that NMJs formed by ESCMNs developed perforations and grew to an average size of ≈120 μm^2^ by 21 days in co-culture. This time course is remarkably consistent with NMJ maturation in the mouse [44], demonstrating the effectiveness of this system to study late events in synapse formation such as the elaboration of AChR clusters and the perforation of plaques into pretzel-like structures.

### Presynaptic NCAM as a regulator of synaptic development and vesicle cycling

One of the most significant findings in the present study was how well *in vitro* NMJs lacking presynaptic NCAM mimicked their endogenous counterparts in *NCAM^-/-^* mice at newly formed endplates [16], [22]. *NCAM^-/-^* mice exhibit structural and functional deficits in NMJ organization, characterized by reduced synapse size, impaired presynaptic vesicle and L-VDCC localization, disrupted neurotransmission, and altered synaptic vesicle recycling mechanisms [16], [20]. All of these phenotypes were observed in our *NCAM^-/-^* ESCMN/myotube co-cultures. Thus, it is tempting to speculate that the previously described phenotypes in the *NCAM^-/-^* mice were due to a lack of presynaptic NCAM rather than its loss post or perisynaptically.

It is becoming increasingly clear that NCAM influences the maturation of presynaptic endocytotic machinery [20], [26], [35]. During axon outgrowth, immature endocytotic mechanisms promote the recycling of synaptic vesicles in the axonal shaft [26], [45]. Axonal recycling can be blocked by applying L-VDCC inhibitors such as nifedipine, or ADP ribosylation factor-1 (ARF1) inhibitors that block adaptor protein 3 (AP-3) mediated endocytosis such as brefeldin A (BFA) [20], [26], [45], [46]. The immature mechanism is eventually down-regulated upon target contact [26], and replaced with an adaptor protein-2 (AP-2) and dynamin-mediated form of endocytosis during synapse maturation [34], [35]. A recent study has demonstrated that AP-2 binds to the intracellular domain of the 140 kD isoform of in an age-dependent manner, and this interaction leads to the displacement of AP-3 at the synaptic membrane in favor of AP-2 [35]. NCAM140 therefore regulates the switch from an AP-3 to AP-2 mediated endocytotic-mechanism by tuning the affinity of the synaptic membrane for adaptor protein interaction.

The link between AP-3 and L-VDCC activity remains to be determined. However, our data demonstrate that acute L-VDCC inhibition completely inhibits FM dye uptake at *NCAM^-/-^* NMJs, but has little effect on synaptic vesicle release, demonstrating a specific effect of L-VDCCs on endocytosis. Chronic inhibition of L-VDCCs led to markedly enhanced neurotransmission and synaptic growth in *NCAM^-/-^* ESCMNs, demonstrating that several NCAM null phenotypes can be rescued by pharmacologically manipulating the immature, AP-3 mediated synaptic vesicle recycling pathway. As AP-2 and AP-3 mediated endocytosis can be dynamically interchanged [35], L-VDCC activity may influence the stoichiometry of AP-2 and AP-3 at the synaptic membrane in favor of AP-3. Inhibition of L-VDCC activity would then shift the affinity of the synaptic membrane away from AP-3 in favor of AP-2, and ultimately produce the same regulatory effect that NCAM would if it was normally expressed.

### ESCMN/myotube co-cultures for the study of MNDs

Motor neurons derived from pluripotent stem cells carrying MND mutations are currently being exploited as tools to study disease pathophysiology and for identifying novel drug targets [47]. This is, in part, because stem cell-derived motor neurons carrying SOD1, TDP-43 or SMN1 mutations exhibit many of the same pathophysiological traits as their endogenous counterparts [41], [48]-[51]. They clearly grow slower and die faster than similarly cultured wild-type motor neurons [49], [50]. The rate of cell death is accelerated when co-cultured with glial cells carrying the same MND mutation [48] or when challenged by trophic factor withdrawal [51]. However, simply recording motor neuron cell death may not be sufficient when screening molecules designed to slow down disease progression. Motor neurons expressing mutant SOD1 withdraw from NMJs prior to cell death [2] and withdraw even when cell death is prevented [4]. It is therefore imperative that this reality is considered when using ESCMNs to model disease progression [52]. The present study suggests that ESCMN/myotube co-cultures, grown as described, reproducibly models disorders of the NMJ. Furthermore, this bioassay can be adapted to study MNDs using motor neurons differentiated from iPS cells derived from individuals with ALS and SMA.

## References

[1] Frey D, Schneider C, Xu L, Borg J, Spooren W, et al (2000) Early and selective loss of neuromuscular synapse subtypes with low sprouting competence in motoneuron diseases. J Neurosci 20: 2534–2542. 10729333 10.1523/JNEUROSCI.20-07-02534.2000 PMC6772256

[2] Fischer LR, Culver DG, Tennant P, Davis AA, Wang M, et al (2004) Amyotrophic lateral sclerosis is a distal axonopathy: evidence in mice and man. Exp Neurol 185: 232–240. 14736504 10.1016/j.expneurol.2003.10.004

[3] Schaefer AM, Sanes JR, Lichtman JW (2005) A compensatory subpopulation of motor neurons in a mouse model of amyotrophic lateral sclerosis. J Comp Neurol 490: 209–219 doi:10.1002/cne.20620 16082680 10.1002/cne.20620

[4] Gould TW, Buss RR, Vinsant S, Prevette D, Sun W, et al (2006) Complete Dissociation of Motor Neuron Death from Motor Dysfunction by Bax Deletion in a Mouse Model of ALS. J Neurosci 26: 8774–8786. 16928866 10.1523/JNEUROSCI.2315-06.2006 PMC6674380

[5] Kariya S, Park G-H, Maeno-Hikichi Y, Leykekhman O, Lutz C, et al (2008) Reduced SMN protein impairs maturation of the neuromuscular junctions in mouse models of spinal muscular atrophy. Hum Mol Genet 17: 2552–2569. 18492800 10.1093/hmg/ddn156 PMC2722888

[6] Kong L, Wang X, Choe DW, Polley M, Burnett BG, et al (2009) Impaired Synaptic Vesicle Release and Immaturity of Neuromuscular Junctions in Spinal Muscular Atrophy Mice. J Neurosci 29: 842–851. 19158308 10.1523/JNEUROSCI.4434-08.2009 PMC2746673

[7] Ruiz R, Casañas JJ, Torres-Benito L, Cano R, Tabares L (2010) Altered Intracellular Ca2+ Homeostasis in Nerve Terminals of Severe Spinal Muscular Atrophy Mice. J Neurosci 30: 849–857. 20089893 10.1523/JNEUROSCI.4496-09.2010 PMC6633088

[8] Imlach WL, Beck ES, Ben Jiwon Choi, Lotti F, Pellizzoni L, et al (2012) SMN Is Required for Sensory-Motor Circuit Function in Drosophila. Cell 151: 427–439. 23063130 10.1016/j.cell.2012.09.011 PMC3475188

[9] Wichterle H, Lieberam I, Porter JA, Jessell TM (2002) Directed differentiation of embryonic stem cells into motor neurons. Cell 110: 385–397. 12176325 10.1016/s0092-8674(02)00835-8

[10] Miles GB, Yohn DC, Wichterle H, Jessell TM, Rafuse VF, et al (2004) Functional Properties of Motoneurons Derived from Mouse Embryonic Stem Cells. J Neurosci 24: 7848–7858. 15356197 10.1523/JNEUROSCI.1972-04.2004 PMC6729934

[11] Soundararajan P, Miles GB, Rubin LL, Brownstone RM, Rafuse VF (2006) Motoneurons Derived from Embryonic Stem Cells Express Transcription Factors and Develop Phenotypes Characteristic of Medial Motor Column Neurons. J Neurosci 26: 3256–3268. 16554476 10.1523/JNEUROSCI.5537-05.2006 PMC6674087

[12] Guo X, Gonzalez M, Stancescu M, Vandenburgh HH, Hickman JJ (2011) Neuromuscular junction formation between human stem cell-derived motoneurons and human skeletal muscle in a defined system. Biomaterials 32: 9602–9611. 21944471 10.1016/j.biomaterials.2011.09.014 PMC3200565

[13] Umbach JA, Adams KL, Gundersen CB, Novitch BG (2012) Functional Neuromuscular Junctions Formed by Embryonic Stem Cell-Derived Motor Neurons. PLoS ONE 7: e36049. 22574134 10.1371/journal.pone.0036049 PMC3344836

[14] Schuster CM, Davis GW, Fetter RD, Goodman CS (1996) Genetic dissection of structural and functional components of synaptic plasticity. I. Fasciclin II controls synaptic stabilization and growth. Neuron 17: 641–654. 8893022 10.1016/s0896-6273(00)80197-x

[15] Schuster CM, Davis GW, Fetter RD, Goodman CS (1996) Genetic dissection of structural and functional components of synaptic plasticity. II. Fasciclin II controls presynaptic structural plasticity. Neuron 17: 655–667. 8893023 10.1016/s0896-6273(00)80198-1

[16] Rafuse VF, Polo-Parada L, Landmesser LT (2000) Structural and functional alterations of neuromuscular junctions in NCAM-deficient mice. J Neurosci 20: 6529–6539. 10964958 10.1523/JNEUROSCI.20-17-06529.2000 PMC6772958

[17] Moscoso LM, Cremer H, Sanes JR (1998) Organization and reorganization of neuromuscular junctions in mice lacking neural cell adhesion molecule, tenascin-C, or fibroblast growth factor-5. J Neurosci 18: 1465–1477. 9454855 10.1523/JNEUROSCI.18-04-01465.1998 PMC6792746

[18] Cremer H, Lange R, Christoph A, Plomann M, Vopper G, et al (1994) Inactivation of the N-CAM gene in mice results in size reduction of the olfactory bulb and deficits in spatial learning. Nature 367: 455–459. 8107803 10.1038/367455a0

[19] Polo-Parada L, Bose CM, Plattner F, Landmesser LT (2004) Distinct Roles of Different Neural Cell Adhesion Molecule (NCAM) Isoforms in Synaptic Maturation Revealed by Analysis of NCAM 180 kDa Isoform-Deficient Mice. J Neurosci 24: 1852–1864. 14985425 10.1523/JNEUROSCI.4406-03.2004 PMC6730389

[20] Polo-Parada L, Bose CM, Landmesser LT (2001) Alterations in transmission, vesicle dynamics, and transmitter release machinery at NCAM-deficient neuromuscular junctions. Neuron 32: 815–828. 11738028 10.1016/s0896-6273(01)00521-9

[21] Polo-Parada L, Plattner F, Bose CM, Landmesser LT (2005) NCAM 180 Acting via a Conserved C-Terminal Domain and MLCK Is Essential for Effective Transmission with Repetitive Stimulation. Neuron 46: 917–931. 15953420 10.1016/j.neuron.2005.05.018

[22] Chipman PH, Franz CK, Nelson A, Schachner M, Rafuse VF (2010) Neural cell adhesion molecule is required for stability of reinnervated neuromuscular junctions. Eur J Neurosci 31: 238–249. 20074227 10.1111/j.1460-9568.2009.07049.x

[23] Rafuse VF, Landmesser LT (2000) The pattern of avian intramuscular nerve branching is determined by the innervating motoneuron and its level of polysialic acid. J Neurosci 20: 1056–1065. 10648711 10.1523/JNEUROSCI.20-03-01056.2000 PMC6774178

[24] Covault J, Sanes JR (1986) Distribution of N-CAM in synaptic and extrasynaptic portions of developing and adult skeletal muscle. J Cell Biol 102: 716–730. 3512581 10.1083/jcb.102.3.716 PMC2114104

[25] Covault J, Sanes JR (1985) Neural cell adhesion molecule (N-CAM) accumulates in denervated and paralyzed skeletal muscles. Proc Natl Acad Sci USA 82: 4544–4548. 3892537 10.1073/pnas.82.13.4544 PMC391139

[26] Hata K, Polo-Parada L, Landmesser LT (2007) Selective Targeting of Different Neural Cell Adhesion Molecule Isoforms during Motoneuron Myotube Synapse Formation in Culture and the Switch from an Immature to Mature Form of Synaptic Vesicle Cycling. J Neurosci 27: 14481–14493. 18160656 10.1523/JNEUROSCI.3847-07.2007 PMC6673458

[27] Arber S, Han B, Mendelsohn M, Smith MA, Jessell TM, et al (1999) Requirement for the homeobox gene Hb9 in the consolidation of motor neuron identity. Neuron 23: 659–674. 10482234 10.1016/s0896-6273(01)80026-x

[28] Gaffield MA, Betz WJ (2007) Imaging synaptic vesicle exocytosis and endocytosis with FM dyes. Nat Protoc 1: 2916–2921. 10.1038/nprot.2006.476 17406552

[29] Rafuse VF, Landmesser LT (1996) Contractile activity regulates isoform expression and polysialylation of NCAM in cultured myotubes: involvement of Ca2+ and protein kinase C. J Cell Biol. 132: 969–983. 10.1083/jcb.132.5.969 PMC2120742 8603927

[30] Chipman PH, Toma JS, Rafuse VF (2012) Generation of motor neurons from pluripotent stem cells. Prog Brain Res 201: 313–331. 23186721 10.1016/B978-0-444-59544-7.00015-9

[31] Sanes JR, Lichtman JW (1999) Development of the vertebrate neuromuscular junction. Annu Rev Neurosci 22: 389–442. 10202544 10.1146/annurev.neuro.22.1.389

[32] Diestel S, Schaefer D, Cremer H, Schmitz B (2007) NCAM is ubiquitylated, endocytosed and recycled in neurons. J Cell Sci 120: 4035–4049. 17971410 10.1242/jcs.019729

[33] Sugiura Y, Ko CP (1997) Novel modulatory effect of L-type calcium channels at newly formed neuromuscular junctions. J Neurosci 17: 1101–1111. 8994064 10.1523/JNEUROSCI.17-03-01101.1997 PMC6573170

[34] Newton AJ, Kirchhausen T, Murthy VN (2006) Inhibition of dynamin completely blocks compensatory synaptic vesicle endocytosis. Proc Natl Acad Sci USA 103: 17955–17960. 17093049 10.1073/pnas.0606212103 PMC1693854

[35] Shetty A, Sytnyk V, Leshchyns'ka I, Puchkov D, Haucke V, et al (2013) The Neural Cell Adhesion Molecule Promotes Maturation of the Presynaptic Endocytotic Machinery by Switching Synaptic Vesicle Recycling from Adaptor Protein 3 (AP-3)- to AP-2-Dependent Mechanisms. J Neurosci 33: 16828–16845. 24133283 10.1523/JNEUROSCI.2192-13.2013 PMC6618524

[36] Fischbach GD (1970) Synaptic potentials recorded in cell cultures of nerve and muscle. Science 169: 1331–1333. 5454145 10.1126/science.169.3952.1331

[37] Chow I, Poo MM (1985) Release of acetylcholine from embryonic neurons upon contact with muscle cell. J Neurosci 5: 1076–1082. 3884749 10.1523/JNEUROSCI.05-04-01076.1985 PMC6564999

[38] Dutton EK, Uhm CS, Samuelsson SJ, Schaffner AE, Fitzgerald SC, et al (1995) Acetylcholine receptor aggregation at nerve-muscle contacts in mammalian cultures: induction by ventral spinal cord neurons is specific to axons. J Neurosci 15: 7401–7416. 7472493 10.1523/JNEUROSCI.15-11-07401.1995 PMC6578065

[39] Takahashi T, Nakajima Y, Hirosawa K, Nakajima S, Onodera K (2002) Structure and physiology of developing neuromuscular synapses in culture. J Neurosci 7: 473–481. 10.1523/JNEUROSCI.07-02-00473.1987 PMC6568910 3029342

[40] Zhang XH, Poo M-M (2002) Localized synaptic potentiation by BDNF requires local protein synthesis in the developing axon. Neuron 36: 675–688. 12441056 10.1016/s0896-6273(02)01023-1

[41] Marteyn A, Maury Y, Gauthier MM, Lecuyer C, Vernet R, et al (2011) Mutant Human Embryonic Stem Cells Reveal Neurite and Synapse Formation Defects in Type 1 Myotonic Dystrophy. Cell Stem Cell 8: 434–444. 21458401 10.1016/j.stem.2011.02.004

[42] Son EY, Ichida JK, Wainger BJ, Toma JS, Rafuse VF, et al (2011) Conversion of Mouse and Human Fibroblasts into Functional Spinal Motor Neurons. Cell Stem Cell 9: 205–218. 21852222 10.1016/j.stem.2011.07.014 PMC3188987

[43] Proszynski TJ, Gingras J, Valdez G, Krzewski K, Sanes JR (2009) Podosomes are present in a postsynaptic apparatus and participate in its maturation. Proc Natl Acad Sci USA 106: 18373–18378. 19822767 10.1073/pnas.0910391106 PMC2775295

[44] Misgeld T, Burgess RW, Lewis RM, Cunningham JM, Lichtman JW, et al (2002) Roles of neurotransmitter in synapse formation: development of neuromuscular junctions lacking choline acetyltransferase. Neuron 36: 635–648. 12441053 10.1016/s0896-6273(02)01020-6

[45] Zakharenko S, Chang S, O'Donoghue M, Popov SV (1999) Neurotransmitter secretion along growing nerve processes: comparison with synaptic vesicle exocytosis. J Cell Biol 144: 507–518. 9971745 10.1083/jcb.144.3.507 PMC2132923

[46] Ooi CE, Dell'Angelica EC, Bonifacino JS (1998) ADP-ribosylation factor 1 (ARF1) regulates recruitment of the AP-3 adaptor complex to membranes. J Cell Biol 142: 391–402. 9679139 10.1083/jcb.142.2.391 PMC2133064

[47] Grskovic M, Javaherian A, Strulovici B, Daley GQ (2011) Induced pluripotent stem cells —opportunities for disease modelling and drug discovery. Nat Rev Drug Discov 10: 915–929. 22076509 10.1038/nrd3577

[48] Di Giorgio FP, Boulting GL, Bobrowicz S, Eggan KC (2008) Human Embryonic Stem Cell-Derived Motor Neurons Are Sensitive to the Toxic Effect of Glial Cells Carrying an ALS-Causing Mutation. Stem Cell 3: 637–648. 10.1016/j.stem.2008.09.017 19041780

[49] Bilican B, Serio A, Barmada SJ, Nishimura AL, Sullivan GJ, et al (2012) Mutant induced pluripotent stem cell lines recapitulate aspects of TDP-43 proteinopathies and reveal cell-specific vulnerability. Pro Natl Acad Sci USA 109: 5803–5808. 10.1073/pnas.1202922109 PMC3326463 22451909

[50] Serio A, Bilican B, Barmada SJ, Ando DM, Zhao C, et al (2013) Astrocyte pathology and the absence of non-cell autonomy in an induced pluripotent stem cell model of TDP-43 proteinopathy. Proc Natl Acad Sci USA 110: 4697–4702. 23401527 10.1073/pnas.1300398110 PMC3607024

[51] Yang YM, Gupta SK, Kim KJ, Powers BE, Cerqueira A, et al (2013) A Small Molecule Screen in Stem-Cell-Derived Motor Neurons Identifies a Kinase Inhibitoras a Candidate Therapeutic for ALS. Stem Cell 12: 713–726. 10.1016/j.stem.2013.04.003 PMC3707511 23602540

[52] Thomson SR, Wishart TM, Patani R, Chandran S, Gillingwater TH (2011) Using induced pluripotent stem cells (iPSC) to model human neuromuscular connectivity: promise or reality? J Anat 220: 122–130. 22133357 10.1111/j.1469-7580.2011.01459.x PMC3275767

